# Getting the Right Traits: Reproductive and Dispersal Characteristics Predict the Invasiveness of Herbaceous Plant Species

**DOI:** 10.1371/journal.pone.0123634

**Published:** 2015-04-23

**Authors:** Lenka Moravcová, Petr Pyšek, Vojtěch Jarošík, Jan Pergl

**Affiliations:** 1 Department of Invasion Ecology, Institute of Botany, The Czech Academy of Sciences, Průhonice, Czech Republic; 2 Department of Ecology, Faculty of Science, Charles University in Prague, Czech Republic; Fudan University, CHINA

## Abstract

To better understand the effect of species traits on plant invasion, we collected comparative data on 20 reproductive and dispersal traits of 93 herbaceous alien species in the Czech Republic, central Europe, introduced after 1500 A. D. We explain plant invasion success, expressed by two measures: invasiveness, i.e. whether the species is naturalized but non-invasive, or invasive; and dominance in plant communities expressed as the mean cover in vegetation plots. We also tested how important reproductive and dispersal traits are in models including other characteristics generally known to predict invasion outcome, such as plant height, life history and residence time. By using regression/classification trees we show that the biological traits affect invasion success at all life stages, from reproduction (seed production) to dispersal (propagule properties), and the ability to compete with resident species (height). By including species traits information not usually available in multispecies analyses, we provide evidence that traits do play important role in determining the outcome of invasion and can be used to distinguish between alien species that reach the final stage of the invasion process and dominate the local communities from those that do not. No effect of taxonomy ascertained in regression and classification trees indicates that the role of traits in invasiveness should be assessed primarily at the species level.

## Introduction

Species traits associated with invasiveness has been a central theme in invasion ecology since biological invasions started to be intensively studied in the 1980s [1]. After 30 years of research, predicting which species will become invasive still represents an ultimate goal of invasion ecologists [2–6]. After the initial period of rather naïve, simple comparisons of species traits [7]; (see [8] for a review), invasion ecologists realized the complexity of the issue and started to address it with sophisticated frameworks [9] and statistical models that take confounding factors and correlative structure of data into account [10–16]. These studies contradicted the view that traits cannot be used for predicting invasion success [17–20]; but see [21, 22] and identified a number of traits that are associated with invasive behaviour by using rigorous comparisons of congeners and meta-analytical approaches [8, 23]. Invasive species were shown to have higher trait means than non-invasive species for traits related to physiology, leaf-area allocation, shoot allocation, growth rate, size and fitness [23]. Other studies identified small genome size [24, 25], seed size [16, 26–28], vegetative reproduction, or ability to flower early as traits linked to invasiveness [8, 29].

However, to address the role of traits in invasions properly, one needs to consider several issues. First, it is now widely accepted that invasions are context-dependent, as is the role of traits in the invasions process and other confounding factors such as propagule pressure [30–34], time since introduction [3, 35, 36], or habitat type [37–39]. Another facet of this context-dependence is the stage the species reaches in the invasion process, viewed as the introduction-naturalization-invasion continuum [5]. Stage-dependence has been demonstrated in tropical systems [40] and in a study using central-European species as a source pool of global plant invasions [15]. In the latter study, the probability of a European species becoming alien outside its native distribution range was determined by the size of its native range and its tolerance of a wide range of climates acquired in the region of origin. Biological traits played only an indirect role at the initial stage of invasion via determining the size of the native range. However, the ability of a species to become an invasive weed was determined not only by the characteristics of native distribution described above, but also directly by species life form and strategy, early flowering, tall stature, sexual reproduction, number of ploidy levels, and opportunistic dispersal by a number of vectors [15]. In addition, traits that confer an advantage at a given stage of the process and in a particular habitat may be neutral or even detrimental at another phase and/or for a different habitat. For example, a recent study has shown that a small genome plays a role in the naturalization stage but does not distinguish naturalized species from invasive [25].

Linked to this context-dependence is the second notion that comparisons of species with different status, in terms of origin and stage of invasion, address different biological questions [8, 10, 41–43], an issue formalized in a framework proposed by [9]. These authors suggest that comparisons of alien species differing in the degree of invasiveness in the introduced range are the most direct test of determinants of invasiveness. This approach has been used in database studies mostly for plants and birds [11–13, 44, 45], but rarely in comparative experiments where it is difficult to find non-invasive congeneric counterparts in the introduced range [8, 23, 46].

Moreover, while the transition from the casual to the naturalization stage (sensu[47])is critical and informative for understanding the mechanisms of invasion [48], most of the knowledge fueling invasion theory comes from studies of the most serious invaders [49]. However, this does not mean that the transition from naturalized to invasive itself is thoroughly studied, partly because the classification of invasive species, as opposed to naturalized but not invasive, is rather vague and differs among authors and regions [50]. In the literature it is common that all alien species, including those that are not naturalized and only occur as casuals, are analyzed together. However, lumping truly naturalized species with those that fail to establish following introduction creates a heterogeneous control group in terms of species traits should the characteristics of invasive species be identified. In the present study we thus carried out a more focused comparison by addressing the naturalization-invasion transition, which we approached by comparing invasive species with naturalized but not invasive species, making use of a standard classification of the alien flora of the Czech Republic [51, 52] based on well-defined, rigorous criteria [41, 47].

The third issue to consider when attempting to define a suite of traits favouring invasiveness is that comparative multispecies studies that search for valid general principles across large taxonomic groups are constrained by the absence of data on some traits important for sexual reproduction, such as fecundity, seed production or seed and germination characteristics, yet it is these traits that are highlighted as important in some studies [16, 26]. On the other hand, studies using those rarely available traits are also usually constrained by a limited number of species [8, 53]. The present paper uses a comparative ecological approach [54] with the aim of diminishing the gap between the two approaches mentioned above, i.e. ecological studies of individual invasive species vs multispecies analyses of alien plants. These data were previously analyzed to find whether there are differences in trait values between invasive and naturalized non-invasive species in single-trait analyses. Invasive species significantly differed from naturalized non-invasive species in propagule characteristics, fecundity, dynamics of seedling emergence, and capacity for dispersal [55]. However, single trait analyses cannot identify pure effects of individual traits unbiased by correlation with or mediated by other traits because of the cross-correlation structure of data and absence of control for confounding factors in the models.

Here, following the above rationale and using data collected for this purpose, we test the role of biological traits of plant species alien to the flora of the Czech Republic and ask what are the factors that determine whether a species becomes invasive following introduction or does not become invasive and persists in the naturalization stage. In addition, since invasion success is a multifaceted phenomenon, we also explore the effects of traits on another characteristic, namely the ability of the species to become dominant in invaded communities. First, we ask if reproductive and dispersal traits alone provide a statistically sound explanation of invasion success. Second, since using only reproductive and dispersal traits may provide biased results for the reasons articulated above, we also test whether reproductive and dispersal traits remain predictive when non-reproductive characteristics generally known to predict invasiveness, such as plant height, life history and residence time, are also included in the models. In our models, we consider not only trait means but also variation in traits to assess the possible role of phenotypic plasticity [43, 56, 57]. We also include the effect of taxonomic affiliation of individual species on the patterns explored.

## Materials and Methods

### Species in the dataset

The species set consisted of 93 herbaceous neophytes (alien species introduced to the country after 1500 A.D.; see [55] for the list of species) in the Czech Republic. The species were classified according to their invasion status, using the classification in Pyšek et al. [51, 52]. Of the species set, 41 neophytes were invasive and 52 were not invasive; this subset is further referred to as “naturalized”, meaning naturalized but not invasive (*sensu*[41, 47, 58, 59]). Naturalized species sustain self-replacing populations in the wild and are permanent members of the local flora; invasive species are a subset of naturalized species that currently spread over long distances [58, 59]. The species we studied were previously classified according to these criteria in a catalogue of alien flora of the Czech Republic [51, 52]. The classification of species into these two groups, invasive vs naturalized but not invasive, was explained by the explanatory variables defined below; these variables were also used to explain the other measure of invasion success, i.e. the dominance of the invading species in plant communities.

The 93 species were selected to provide a representative sample of naturalized and invasive species in the Czech flora. They represent 44.9% of the total number of 207 naturalized herbaceous neophytes in the Czech Republic [52] and belong to 70 genera and 32 families according to the Angiosperm Phylogeny Group classification [60]; (see [55] for more details on the data set structure).

### Measures of invasiveness: response variables

Invasion success of the species was expressed by using two measures: (1) *invasiveness*, i.e. whether the species is naturalized but non-invasive, or invasive in the Czech Republic (categorical variable) and (2) *dominance* expressed as the average percent cover in plant communities in the Czech Republic, taken from the Czech National Phytosociological Database of phytosociological relevés [61, 62], based on 62,539 relevés (available for 67 species—both invasive and naturalized, range 6–5,171 relevés per species).

### Species traits: explanatory variables

The 20 species traits used to explain invasion success of the neophytes were (i) “reproductive and dispersal traits” measured for the majority of species at three localities in the field and at the experimental site of common garden of the Institute of Botany in Průhonice in the Czech Republic between 2005–2007 (see[55] on details of sampling, and [25] for the list of localities), including some additional characteristics of species’ reproduction taken from the CzechFlor database held at the Institute of Botany, The Czech Academy of Sciences, and (ii) some additional traits known to affect invasiveness based on previous reviews and studies [8, 15], further referred to as “other traits”. The traits measured were selected to include those that play an important role in plant reproductive behavior and thus likely affect invasion success of the species. Further, they were selected with an aim to encompass the whole reproductive cycle, from (i) seed production to (ii) dispersal and to (iii) establishment. If not stated otherwise, details on methods used to obtain values for particular variables as well as average values for individual species are given in ([55]; their Tables one and two).

#### Seed production traits

Two measures were used to describe the number of generative propagules produced at the scale of individual plants (or shoots) and at the scale of populations: (1) *plant propagule number (fecundity)* is the average number of propagules per single plant, or single shoot of a clonal species (hereafter termed plant). The number of propagules was based on measurements of 10 plants from each population wherever possible. (2) *Population propagule number (seed production)* is an average number of propagules per m^2^ of maximum population density found in the locality. This density was estimated by calculating the number of plants in 10 plots of 1 m^2^, selected in dense stands of the species, to obtain a measure of maximum reproductive capacity of the population. These traits were complemented with additional characteristics taken from the CzechFlor database: (3) *length of flowering period* (in months), (4) *reproductive system* (strictly allogamous species vs species capable of autogamy) and (5) *vegetative reproduction* (species reproducing exclusively by seed vs species capable of vegetative reproduction). We also included (6) *genome size* as a characteristic related to the number and size of the seeds produced by a plant species [63, 64]; these data were taken from [25].

#### Dispersal traits

This includes traits related to propagules that are effectively dispersed, i.e. seed or fruit, depending on species. Measurements were based on 25, 50 or 100 propagules, depending on species, from each locality, and included: length as a measure of (7) *propagule size* (mm), length/width ratio as a two-dimensional measure of (8) *propagule shape* (with low values characterizing rounded propagules); and (9) *propagule weight* (g) obtained by weighing four sets of 25 propagules from each locality, randomly collected from the whole population, and calculating the average weight of one set. (10) *Water dispersal* (buoyancy) was measured for each species using 100 randomly selected propagules from each locality placed in beakers (4 replicates of 25 propagules) filled with distilled water. The time when all propagules sank (Ft_100_, in hours) was used as a measure of buoyancy. (11) *Animal dispersal* (*epizoochory*) was tested as the ability of propagules to attach to wild boar fur. Wild boar was selected because most plant species’ propagules easily attach to its fur, and it can be assumed, due to its migration potential, to spread propagules over a large distance [65]. The number of attached propagules was counted after three circular movements of a frame pressed to the paper. For each species, 100 propagules from each locality were randomly selected and used to make 4 replicates of 25 propagules. To assess the potential for (12) *wind dispersal* (*anemochory*), terminal velocity (m/s) was used, measured by using a special instrument constructed according to [66]; this device makes it possible to measure the speed of a diaspore falling to the ground in standardized conditions which is then used to infer about the distance it can travel airborne. For each species 50 randomly selected diaspores from each locality were used. The value of 1/terminal velocity was used in models so that a higher number suggests better capacity for wind dispersal, reflecting lower speed of seed fall.

#### Establishment traits

Germination was assessed using seeds that were freshly harvested, dry-stored for one month or cold-stratified and then germinated under different temperature regimes (see[55] for details). Each treatment consisted of 4 replicates of 25 seeds. (13) *Maximum germination* (%) is the highest germination percentage achieved by the species under the best of all the germination treatments used. The proportion of seed germinated immediately after harvest, of the total number germinated, was used as a measure of (14) *dormancy*. (15) *Seedling relative growth rate* (RGR, g.g^-1^.day^-1^) was measured following [67] and calculated following [68]. Seedling establishment was measured in a common garden at the Institute of Botany of The Czech Academy of Sciences, with 25 propagules sown in a plastic container 10 × 10 cm in size and for each species, using 3 replicates from each locality. Emerging seedlings were counted in the autumn following sowing and in the spring of the next year. This yielded two measures of all established seedlings: (16) *autumn seedling establishment* is the percentage recorded in the autumn following sowing, (17) *total seedling establishment* is the percentage of seed that appeared by the following spring.

#### Variability of traits

Where applicable, i.e. where obtained values were based on multiple measurements, we calculated coefficient of variation (CV) as a measure of variation in traits. Samples from individual plants and localities were pooled, meaning that all values for a given trait obtained for a species were used in calculation of CV. The measure of variation was obtained for the following traits: propagule length, shape and weight, water dispersal, animal dispersal, wind dispersal and maximum germination. All traits for which CV is given were measured over the same populations within each species sampled (see [25] and their Appendix 1 for the list of localities).

#### Other factors

To account for other species characters that are generally known to affect invasion success and that often appear as significant factors in database analyses focusing on the role of species traits in plant invasions, we included species’ (18) *life history* (annual, monocarpic perennial, polycarpic perennial), (19) *height* (m), and (20) *minimum residence time* (yrs). The residence time, i.e. how long the species has been present in the Czech Republic, was included because it is closely related to various measures of invasion success [3, 69] and if its effect is not considered, comparison of species with different residence times will bias the results [5]. The data on these traits were taken from the CzechFlor database held at the Institute of Botany, The Czech Academy of Sciences, and from [51, 52].

#### Species relatedness

To assess the role of species relatedness on invasion success, taxonomic affiliation of species was used as a predictor.

### Statistical analysis

#### Response and predictor variables

Two models with different response variables describing invasion success were established: Model I with the binary response “invasive/non-invasive” characterizing species invasiveness (n = 93); and Model II with the continuous response variable “percent cover” (n = 67) characterizing species dominance. Because more data give more precise results on percentages [70] to avoid undue influence of small datasets, percent covers in Model II were weighted by the number of relevés from which the cover for each species was calculated. Predictor variables for these models were (a) reproductive and dispersal traits and (b) other traits, as listed above. Models I-IIa included only reproductive and dispersal traits, while Models I-IIb included also other traits. Finally, (c) variability of traits was added as coefficients of variance corrected for sample size [71] to models including both groups of traits, (a) and (b), and analyzed as Models I-IIc. All models also included as predictors the taxonomic affiliation of individual species (Genus, Family, Order, Class).

#### Data exploration

All models were examined by regression and classification trees [72–74]. If the response variable was categorical (Model I), the method was classification trees; if continuous (Models II), regression trees. The data mining techniques enable us to identify the most important predictors by screening a large number of explanatory variables, without requiring any assumptions about the form of the relationships between predictors and the response variable, and without a priori formulated hypotheses [75]. The methods are more flexible than traditional statistical analyses because they can reveal structures in the dataset that are non-linear and to accommodate complex interactions [76]. Because the analysis retains all correlated variables the ranking of the various predictor variables importance thus guards against the elimination of variables that are good predictors of the response, and may be ecologically important, but are correlated with other predictors [77].

Regression and classification trees were calculated in the commercial statistical software (CART Pro v. 6.0 [72–74, 78]). The data were successively split along coordinate axes of predictors using binary recursive partitioning so that at any node, the split was selected that maximally distinguishes the response variable in the left and the right branches, based on Gini impurity measure [73, 77]. In classification trees, all analyses were conducted with balanced class weights [74], assuring that the binary invasive/non-invasive status was treated as equally important for the purpose of achieving classification accuracy. Ten-fold cross-validations were used to obtain optimal trees with the smallest cross-validated errors for interpretation. This cross-validation involved splitting the data into a number of smaller samples with similar distributions of the response variable. Trees were then generated, excluding the data from each subsample in turn. For each tree, the error rate was estimated from the subsample excluded in generating it and the cross-validated error for the overall tree was then calculated [72, 73].

We decided when a tree was complete by growing the tree until it was impossible to grow it further and then examining smaller trees obtained by gradually decreasing the size of the maximal tree [72]. A single optimal tree was then determined by testing for error rates for the largest tree as well as for every smaller tree, with cross-validation used to obtain estimates of relative errors of these trees. These estimates were then plotted against tree size, and the optimal tree chosen both based on the minimum cost tree rule, which minimizes the cross validated error (the default setting in CART v. 6.0; [78]), and based on the one-SE rule, which minimizes cross-validated error within one standard error of the minimum [72]. A series of 50 cross-validations were run based on each rule, and the modal (most likely) single optimal tree chosen for description [76]. The optimal tree with the smallest error was then used for final interpretation, and represented graphically, with the root standing for undivided data at the top, and the terminal nodes, describing the most homogeneous groups of data, at the bottom of the hierarchy.

To prevent variables with missing values having an advantage as splitters, the predictor variables were penalized in proportion to the degree to which they had missing values [78]. Missing cases were treated by back-up rules based on surrogates of each split. These surrogates describe splitting rules that closely mimic the action of the missing primary splitters [72, 78].

#### Measures of predictor importance and overall quality of optimal trees

Effects of individual traits in the optimal trees were expressed by improvement values, corresponding to the proportion of the total variance explained by the trait at each split at each node of a tree [78]. These values were summed for each trait over each node and totalled, and scaled relative to the best-performing trait. The trait with the highest sum of improvements was scored 100, and all other traits had lower scores ranking downwards towards zero. The resulting relative importance scores thus provided a relative measure of each trait’s contribution to the model’s predictive power.

Unlike traditional linear models, the trees cannot properly handle nested designs such as hierarchical taxonomic levels [79]. To overcome the problem that related organisms can have a similar effect [80], the splitting power of taxonomic levels in regression and classification trees was penalized proportionally to the number of categories at each taxonomic level (Genus 70, Family 32, Order 20, Class 2 categories), following penalization rules for high category variables [73].

The overall quality of the chosen optimal classification trees was evaluated by their misclassification rate by comparing it with the misclassification rate of the null models with 50% misclassifications [76, 81, 82]. The quality of optimal regression trees was expressed similarly to total variance R^2^ explained in linear models. For regression trees, it was calculated as R^2^ = 1—resubstitution relative error [74, 76].

## Results

### The effect of taxonomy

In the classification/regression trees, which enabled us to take species relatedness hierarchically into account, taxonomy never appeared as an important predictor scaled more than 10%. Only genus, and in one case family, appeared as a surrogate of primary splitters but their improvement values never reached more than 9.2% of the improvement values of the primary splitters.

### Invasiveness

**Fig 1:**
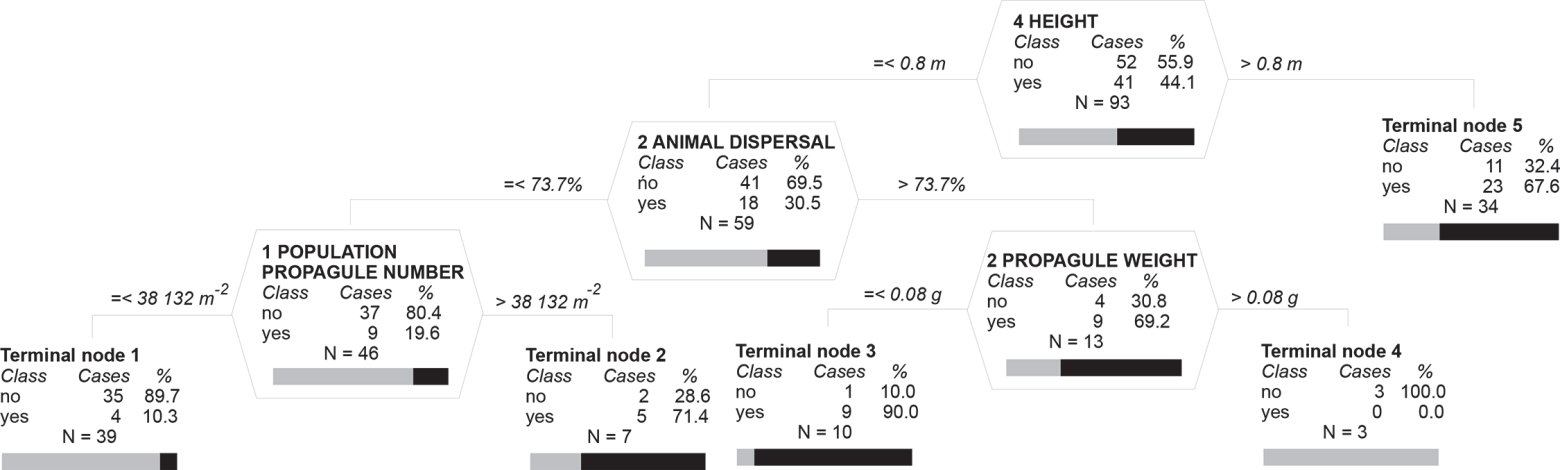
Optimal classification tree of the probability of a plant species being invasive (yes ■) or naturalized but not invasive (no □) for model including all traits (Model Ib in Table 1). Each node (polygonal table with splitting variable name) and terminal node (with node number) shows table with columns for invasiveness (Class no or yes) and number (Cases) and percent (%) of cases for each Class. Below the table is the total number of cases (N) and graphical representation of the percentage of no and yes cases in each Class (horizontal bar). For each node, there is a split criterion on its left- and right-hand side, rounded to one decimal point. Vertical depth of each node is proportional to its improvement value that corresponds to the explained variance at the node. See Table 1 for overall misclassification rate of the optimal tree.

All models identified significant predictors of invasiveness (Table 1). In the classification tree with only reproductive and dispersal traits included (Model Ia), the probability of species being invasive depended solely on the population propagule number, reaching 78.9% when populations produced more than 37,700 propagules per m^2^, but only 36.1% when it produced less. The inclusion of other traits yielded a more structured Model Ib with plant height, animal dispersal (epizoochory) and propagule weight adding to population propagule number in determining whether a species was invasive or not (Table 1 and Fig 1). Species taller than 0.8 m had 67.6% probability of being invasive (terminal node 5 in Fig 1). Shorter species were most likely to be invasive if they were capable of good animal dispersal (more than 73.7% of seed attached to the wild boar fur, see Data) and had seeds lighter than 0.08 g; 90% of species with this suite of traits were invasive (terminal node 3). Invasiveness of species with lower capacity for animal dispersal was determined by the number of propagules produced; 71.4% of species whose population propagule number exceed 38,100 per m^2^ were invasive (terminal node 2), but only 10.3% of those with population propagule number below this threshold (terminal node 1). Including variation in traits (Model Ic) did not affect the results; variation in none of the traits had a significant effect on the predictors of invasiveness.

**Table 1:** Effect of species traits on invasiveness (invasive or naturalized but non-invasive; Models I), and dominance (percent cover in invaded communities; Models II) of alien herbaceous plants.

Model / Response variable	I-IIa: Reproductive/dispersal traits		I–IIb: Reproductive/dispersal + other traits		I–IIc: Reproductive/dispersal + other traits+ variation	
	Trait	Importance (%)	Trait	Importance (%)	Trait	Importance (%)
I. Invasiveness	1 Population propagule number	100.0	4 Height	100.0	3 Height	100.0
			2 Animal dispersal	89.0	2 Animal dispersal	89.0
			1 Population propagule number	80.1	1 Population propagule number	80.1
			2 Propagule weight	73.1	2 Propagule weight	73.1
Misclassification rate (%)	32.3		22.6		22.6	
II. Dominance			3 Height	100.0	3 Height	100.0
Explained variance (%)	NS		32.7		32.7	

Reproductive and dispersal species traits (Models a) are classified by whether they are related to seed production (coded 1 before the variable name), or dispersal (coded 2). Other traits affecting invasion success (coded 3) were added to Models b, and variation in traits to Models c (see text for details). Effects are expressed as importance relative to the most important predictor (%), and the overall significance of each model as percent of misclassifications compared to null model with 50% misclassification rate (Models I) and percent of explained variance (Models II); better models have lower misclassification rate in classification trees (Models I) and explain more variance in regression trees (Models II).

### Dominance

Regression trees only selected predictors of species dominance, expressed as the percentage cover in plant communities, if the model included both reproductive/dispersal and other traits (Model IIb in Table 1). In this model, the dominance was affected only by plant height. The average cover of the naturalized species was 7.4%±4.3 (±standard deviation; n = 58) if the species was shorter than 1 m but 17.6%±9.2 (n = 9) if it was taller. The inclusion of variation did not change this result (Model IIc).

## Discussion

### Searching for determinants of invasion success: getting the right traits and eliminating confounding factors

A different research avenue than our approach is analyses of regional inventories of alien plants that have the potential to produce generalizations over a wide range of taxa, usually over vascular plants as a whole [26, 83, 84]; (see [8] and [53] for reviews). A major constraint of such multispecies analyses is the rather limited information on plant characteristics used as predictors of invasive behaviour. Most comparative papers use available data from databases in which the information on complete floras is of limited comparability because it was collected by many workers using various methods, and information on some traits is not available [6]. Among data that are most urgently missing is information on reproductive characteristics that are generally considered as important determinants of invasive behaviour [8]. Using selective data only on those traits for which the information is available, however, brings about the danger of yielding biased results and overestimating a factor’s importance, which could disappear in a later analysis if better data were collected.

This paper attempts to overcome this shortcoming by considering information on a number of reproductive and dispersal traits collected by comparable ecological methods for a large number of naturalized and invasive alien species, and using statistical analyses that allow to find among the explanatory variables the most important ones irrespective of their possible collinearity. This is necessary to account for mutual correlation of reproductive factors reflecting well-known demographic relationships such as the trade-off between, e.g., propagule size and fecundity [85–87] or between RGR and seed size [88–90].

Of the two most often highlighted confounding factors that can prevent studies from evaluating the role of species traits properly, are propagule pressure and minimum residence time; the latter was directly included as a variable in our models. Residence time has been repeatedly shown to correlate closely with the current distributions of alien species [91–93] as well as with the species invasion status [69]. Therefore, comparing traits of species with different residence times may yield biased results because the species differ in the period of time provided to realize their colonization potential and reach their full distribution and habitat occupancy in the new range [94].

We did not include an explicit measure of the second most important confounding factor, propagule pressure [30–34, 95–97], into our models because at this scale it is not possible to measure it or obtain reasonable surrogates for individual species over historical periods of time [5]. However, we argue that propagule pressure is implicitly included in some of our variables (population propagule number and residence time). This is because we compared naturalized non-invasive species with invasive species (i.e. all species in our data set were naturalized), and for naturalized species it is likely that the main part of the overall propagule pressure is via their fecundity and seed production in wild populations [91], the characteristic that we measured in the field. The more fecund a naturalized species is the more propagules are spread in the landscape, ready to enter the dispersal phase, and the less it needs to rely on introduction of propagules by human-related pathways [98]. The time factor is implicitly included in the residence time since residence time represents another dimension of propagule pressure; the longer the species is present, the more propagules enter the environment [64]. Therefore, within the same species, its populations release more propagules into the environment if the species is present for a long time in the invaded region than would be the case if the same species was present for a short time. We suggest that this could be the reason why residence time had, unlike in previous studies, no effect on the measures of invasion success in our data, as it was overwhelmed by the effect of population propagule number, and acted as a surrogate for fecundity traits in Fig 1. It also indicates that the predictive power of the variables used in our model is good enough to overcome the effect of varying residence times of the species tested.

Finally, it needs to be noted that another factor influencing the outcome of invasion, i.e. the habitat in which particular species occur [37–39], could not be quantitatively tested in our analyses for logistic reasons. To do this properly would require to sample species across the whole range of habitats they occupy. However, the species included in the analysis are all successful invaders in the Czech flora and the majority of them occur in a wide range of habitats [99]; therefore, we sampled the traits of each species at three sites representing habitats the species typically inhabit, to account for the variation in the range of field conditions.

### Species traits associated with invasiveness: grow, beget and disperse

Unless rigorously defined, “invasion success” remains a rather vague multi-layered term composed of various measures of species’ population growth and dispersal determining its spatio-temporal dynamics and position along the introduction-naturalization-invasion continuum [47, 58]. This notion is emphasized by the fact that different factors play different roles at various stages of the invasion process; it has been suggested that social and economic factors are crucial at the introduction stage, biogeographical and ecological factors at the stage of naturalization, and ecological and evolutionary principles are crucial at the stage of invasion [100, 101]. From this it follows that the role of ecological traits is most sensible to examine for transitions between introduction and naturalization [48, 49], and between naturalization and invasion as in the present study.

Although fecundity per plant, as an intrinsic species attribute, turned out in previous studies to be sufficient to explain invasion success in some groups of plants [102] and was also shown to account for a difference between widespread and narrow endemic species [103], our results suggest that measurements of seed production at the population level make a more robust predictor than those for an individual plant. The population propagule number, which represented the potential fecundity of the individual species, was the only significant predictor of its probability of being invasive identified by the classification trees that were run on reproductive and dispersal traits only. However, including the other traits known to affect the outcome of invasion yielded a more structured model that provides a deeper insight into the determinants of invasiveness of central-European herbaceous alien plants. Species biological traits that determine whether a species becomes invasive or remains naturalized but non-invasive (in the sense of [47, 59]) include height, animal dispersal, seed production and propagule weight. Species taller than 0.8 m are likely to be invasive without other specification; this height roughly corresponds to the upper vegetation layer of herbaceous communities in the temperate zone [104] and indicates the threshold for competitively strong dominants [105, 106, 64]. Shorter species can compensate by efficient dispersal by animals, attached to fur; if their seeds are small, they are the most successful group with 90% probability of achieving invasion status. Shorter species with less efficient animal dispersal can still be invasive provided that they compensate for these disadvantageous traits by producing a large number of propagules. We believe that this model reasonably well characterizes the factors determining invasiveness of central-European herbaceous alien plants; the results do not depend on taxonomy, as it was ascertained in regression and classification trees. This points to previously reported findings that assessments of the role of traits in invasion success and that of the risk of invasion should be made primarily at the species level and that generalizations based on higher taxonomic levels can be misleading [15, 107]. Still, it needs to be borne in mind that the number of species for which we measured the reproductive and dispersal characteristis is still rather low compared to hundreds of species used in macroecological studies of the whole floras (see e.g. [8] for review), which somewhat limits the general validity of our results. Nevertheless, our results not only point to that the reproductive and dispersal traits are important for invasion success of plant species but also suggest that it is crucial to collect comparative and precise data on these traits for more species from different regions and environments.

Phenotypic plasticity has been shown to have an important effect on invasiveness and many studies demonstrated that invasive plants show greater levels of plasticity than their native or non-invasive congeners [57,108–111]. That in our study the plasticity did not have a significant effect on separating invasive species from non-invasive can be, however, related to the way how it was assessed, with the coefficient of variation being rather crude proxy compared to measurements performed in studies that found the important effects of plasticity. This points to the recent suggestion that experiments over realistic range of environmental conditions are needed [112].

For the invasion of plant communities at the local scale of vegetation plots (tens to hundreds of square meters in size), the only trait that contributes to achieving a high cover is plant height; taller plants are more likely to become dominants in resident communities. This corresponds to the role that invader’s stature plays in central-European herbaceous vegetation, where the strength of impact on species diversity of invaded communities results from the interaction between the height of the invader and that of native dominants [113,114], and to the fact that plant height is closely related to a species’ competitive ability [115]. It needs to be, nevertheless, pointed out that the informative value of this particular analysis is somewhat limited due to the fact that data on cover in plant communities at the scale of vegetation plots were only available for about two thirds of species (n = 67) analysed. Also, we did not measure quantitatively the vigour of spatial growth associated with vegetative reproduction, which is considered an important prerequisite for space preemption; this trait has been previously shown to contribute to invasiveness of plant species [8].

The importance of having quantitative data on crucial reproductive traits can serve as an explanation of why, for example, life history or life strategy, often highlighted as characteristic determining invasion success [15, 27, 83], (see [8] for a review), do not appear significant in the present study. This is because life history/strategy is a synthetic characteristic largely defined by traits that are included here separately (seed size, fecundity, vegetative vs generative reproduction, seed survival etc. [54, 115]). This is not to undermine the value of life history as an important variable in risk-assessment schemes [116], but to point out that more meaningful results can be obtained if precise locally collected data is available.
